# Silver Nanoparticles Coated Poly(L-Lactide) Electrospun Membrane for Implant Associated Infections Prevention

**DOI:** 10.3389/fphar.2020.00431

**Published:** 2020-04-08

**Authors:** Jiaolong Wang, Lilin Zhan, Xianhua Zhang, Runfa Wu, Lan Liao, Junchao Wei

**Affiliations:** ^1^School of Stomatology, Nanchang University, Nanchang, China; ^2^The Key Laboratory of Oral Biomedicine, Nanchang, China; ^3^College of Chemistry, Nanchang University, Nanchang, China

**Keywords:** poly(L-lactide), electrospun, antibacterial, Ag nanoparticles, guided bone regeneration

## Abstract

Bacterial infection has been a critic problem for implant infections. Poly(L-lactide) (PLLA) membrane has great potential for Guided bone regeneration (GBR), however, PLLA lack antibacterial property and thus may face bacterial infections. In this work, a mussel inspired method was used to treat PLLA membrane with dopamine and formed polydopamine (PDA) coated PLLA (PLLA@PDA), and then silver Nanoparticles (AgNPs) was immobilized on the surface of PLLA *via* the reduction effect of PDA. The XPS results showed that the silver element contents may be tuned from 1.6% to 15.4%. The AgNPs coated PLLA (PLLA@Ag) showed good antibacterial property (98.3% bactericidal efficiency may be obtained) and good biocompatibility, implying that the PLLA@Ag membrane have potential application as antibacterial GBR membrane, which may enhance the application of PLLA.

## Introduction

Guided bone regeneration (GBR), is a common and promising augmentation technique to regain sufficient width and height of the jawbone at oral implant sites, and has been an important surgical procedure to heal peri-implantitis defects (Lundgren et al., 1994; Wang et al., 2016; Spinell et al., 2019). A critical factor that determines the success of GBR technique is the GBR membrane, which can prevent epithelial or undesirable tissues migration into the defective area (Wang et al., 2016). Up to now, various materials, such as collagen, polytetrafluoraethylene, have been widely used as GBR membrane. Polylactic acid, an important biodegradable polymer, due to its good biocompatibility, suitable mechanical strength, and controllable degradation, has been approved by Food and Drug Administration (FDA) and used on clinic (Santoro et al., 2016; Tyler et al., 2016; Hamad et al., 2018), showing great potential as GBR membrane (Wang et al., 2016).

Bacterial infection has been a common headache in our daily life or clinics. Bacterial adhere on the surface of medical materials, resulting in infections or even failure of materials or surgery operation (Chen et al., 2016; Behzadi et al., 2017; Jensen et al., 2017; Shen et al., 2019), as for GBR technique, bacterial infection has been a major reason for failure of GBR *in vivo* (Slutzkey et al., 2015). And thus, it is definitely needed to develop an effectively antibacterial GBR membrane, which can regain sufficient bone, and reduce bacterial infections (Shi et al., 2019; Wang et al., 2019). Recently, GBR membranes with antibacterial activity have been prepared by the addition of antimicrobial agents, such as doxycycline (Kutan et al., 2016), metronidazole (Xue et al., 2014a; Xue et al., 2014b), chlorhexidine (Qian et al., 2018), and so on, showing great potential for bone regeneration. However, antimicrobial resistance has limited the applications of these organic antimicrobial agents, as Rams et al. Reported, 71.7% of 120 peri-implantitis patients appeared resistance to one or more antibiotics (Rams et al., 2014). Therefore, it is essential to develop GBR materials with alternative antibacterial agents.

Owing to the broad-spectrum antibacterial and few drug resistance properties, silver nanoparticles (AgNPs) have been widely used as antibacterial agent or surface coating in dental instrument, contact lenses, cardiovascular stent, implant, et al. (Le Ouay and Stellacci, 2015; Qiao et al., 2019; Shakya et al., 2019). However, during the synthesis process, toxic reducing agent are always used, which may increase the cytotoxicity or potential risk of environment pollution (Duan et al., 2015). Hence, it is essential to design a novel, ecosystem friendly way to prepare AgNPs loaded antibacterial GBR membranes.

Inspired by the adhesion property of mussel, polydopamine (PDA) has been coated onto various surfaces *via* the self-polymerization of dopamine (Lee et al., 2007; Xin et al., 2018). PDA contains many catechol, amino, and quinone groups, which can be used to connect other molecules or functional particles on PDA treated surfaces (Huang et al., 2017; Lin et al., 2017). Furthermore, due to the reduction effect of catechol groups, AgNPs can be synthesized *via* the in-situ reduce reaction of silver ion, and thus this mussel inspired method have been widely used to immobilize AgNPs on various matrix, such as cellulose paper (Islam et al., 2018), mesoporous silica (Song et al., 2018), and polymer electrospun fibers (Wang et al., 2017).

Although PLLA has showed many potential applications as GBR membrane, it is much important to prepare antibacterial PLLA, which can prevent the implant associated infections. In this work, poly(L-lactide) (PLLA) electrospun nanofibers membrane was prepared as a GBR membrane model. Mussel inspired method was used to prepare AgNPs coated PLLA membranes (PLLA@Ag), which can combine the antibacterial property of AgNPs and bioproperties of PLLA. The modified PLLA membranes showed superior antibacterial property and good biocompatibility, showing great potential for clinic application as GBR membrane.

## Materials and Methods

### Materials

Poly(L-lactic acid) (HISUN Biological material co., LTD, PLLA revode 190), Dopamine Hydrochloride (purity 98%) (Aladdin Industrial Corporation), silver nitrate (Sinopharm Chemical Reagent co., LTD), Tris (hydroxymethyl) aminomethane (Sinopharm Chemical Reagent co., LTD), Tris (hydroxymethyl) aminomethane hydrochloride (AlfaAesar Chemical Reagent co., LTD), Cell Counting Kit-8 (CCK-8) (Beijing Zoman Biotechnology Co.,Ltd.), and all other materials used in this work were used as received, without further purification.

### Preparation of PLLA Nanofiber Membrane

PLLA membranes were prepared *via* the electrospinning method. Briefly, PLLA was dissolved in volume ratio of 4:1 chloroform/N, N-dimethyl formamide (DMF) mixture under stirring for 24 h at room temperature to obtain a homogeneous electrospinning solution. The electrospinning using the following parameters: cylinder collector speed of 300 rpm, fixed spinning distance of 20 cm, flow rate of 1.2 ml/h, and spinning voltage for 18 KV. The obtained membranes were collected at room temperature and then dried under vacuum for at least 48 h to remove the remained solvent completely.

### Preparation of AgNPs-Loaded PLLA Nanofiber Membrane (PLLA@Ag)

PLLA@Ag was prepared by a simple *in situ* reduction method. Briefly, square PLLA membranes (1 cm X 1 cm) were immersed into the 10 mL dopamine Tris-HCl buffer solution (pH = 8.5, 2mg/ml) for 24 h, followed by washing twice with water and drying under vacuum. Then these PLLA nanofiber membranes with PDA grafted on their surface (PLLA@PDA) were immersed into 10 ml silver nitrate solution (25 mmol/L) for different time intervals (1 h, 3 h, 6 h, 9 h, 24 h), washed and dried, resulting in PLLA nanofiber membrane loaded with different amount of AgNPs (PLLA@Ag). The corresponding membranes were denoted as PLLA@Ag1, PLLA@Ag3, PLLA@Ag6, PLLA@Ag9, and PLLA@Ag24.

### Characterization

The morphology of electrospun membranes were observed with a field-emission scanning electron microscope (FE-SEM, JSM -6701F, JEOL, Japan). X-ray photoelectron spectrometer (XPS, ESCALAB 250, Thermo-fisher, USA) was carried out to analyse surface elements of membranes. X-ray diffractometer (XRD, D8 Focus, Bruker, Germany) was used to investigate the crystalline phase of membranes. The surface contact angle of the membranes was measured using a static contact angle meter (JC2000A, Powereach, China)

### Antibacterial Activity Test

The antibacterial activity against Staphylococcus aureus (*S. aureus*) (ATCC 25586) of PLLA, PLLA@PDA, and PLLA@Ag samples was tested using agar diffusion assays as well as modified colony counting method. In agar diffusion assays, disk-shaped samples (5 mm in diameter) were prepared and sterilized through ultraviolet irradiation for 1 h (30 min each side), before placing on the cultured blood agar plates. 100 μl aliquot of approximately 3*10^8^ cfu/ml bacterial suspension was spread onto an agar plate. Disk-shaped samples were placed on agar plates (repeat three times for every sample). Then the plates were incubated for different times at 37°C. The bacterial growth on the plate was visualized directly and the diameter of the inhibition zone was measured on days 1 day, 3 days, 7 days, and 14 days.

In modified colony counting method, sections (1 cm × 1 cm) of nanofiber membrane were placed in a sterilized flask containing 100 μl of test organisms and incubated at 37°C for 4 h in a laboratory shaker at 200 rpm. After 4 h incubation, serial dilutions of the liquid were made in PBS. Dilution of 10^4^, 10^5^, and 10^6^ were used for colony counting method. 50 μl of every dilution was spread on to the agar plate and then incubated at 37°C for 24 h. After incubation, the number of viable colonies were counted manually. The percent reduction in number of colonies in PLLA (in their different) treated sample as compared to the untreated samples gives the antibacterial activity of the treated samples.

The bactericidal efficiency of the membranes was calculated by the following formula:

$$\text{Bacteridical efficiency }(\%)=\frac{\text{A}-\text{B}}{\text{A}}*100\%$$

where A is the average number of the colonies of untreated group and B is the average number of the colonies of the treated samples.

### Cytotoxicity Test

Cell proliferation was determined using a cell counting kit (CCK-8) assay. All the nanofiber membranes were cut into squares (1 cm × 1 cm), sterilized under UV light (30 min each side), immersed in 70% ethanol for 30 min, and then washed twice with sterile PBS. The sterile membranes were incubated in 1mL Dulbecco’s modified eagles medium (DMEM) for 48 h at 37°C under a 5% CO2 humidified atmosphere, and then the supernatant was filtered by Millipore^®^ membrane to obtain the extract liquid. The extract liquid was serially diluted into 50%, 25%, 12.5%, and 6.25%, respectively.

Next, osteoblast MC3T3-E1 cells (1×10^4^ cells/ml) were seeded into each well (100 µl/well) and incubated at 37°C under 5% CO_2_ atmosphere for 24 h to make them proliferate and adhere on the wall. And then, 100 μl extract liquid and the corresponding dilutions were added into each well to replace the media, and cultured for 48 h. 20 µl of CCK-8 buffer was added to each well, and cells were incubated at 37°C for an additional 2 h. The absorbance was measured at λ= 450 nm on a plate reader.

Another cell line (fibroblast L929) was also used to do the same experiments according to the above the procedure.

### Statistical Analysis

The obtained data were presented as mean ± SD. Statistics differences were checked by One-Way Analysis of Variance, and the signiﬁcance was set at P < 0.05.

## Results and Discussion

### Characterizations of Nanofiber Membranes

**Figure 1:**
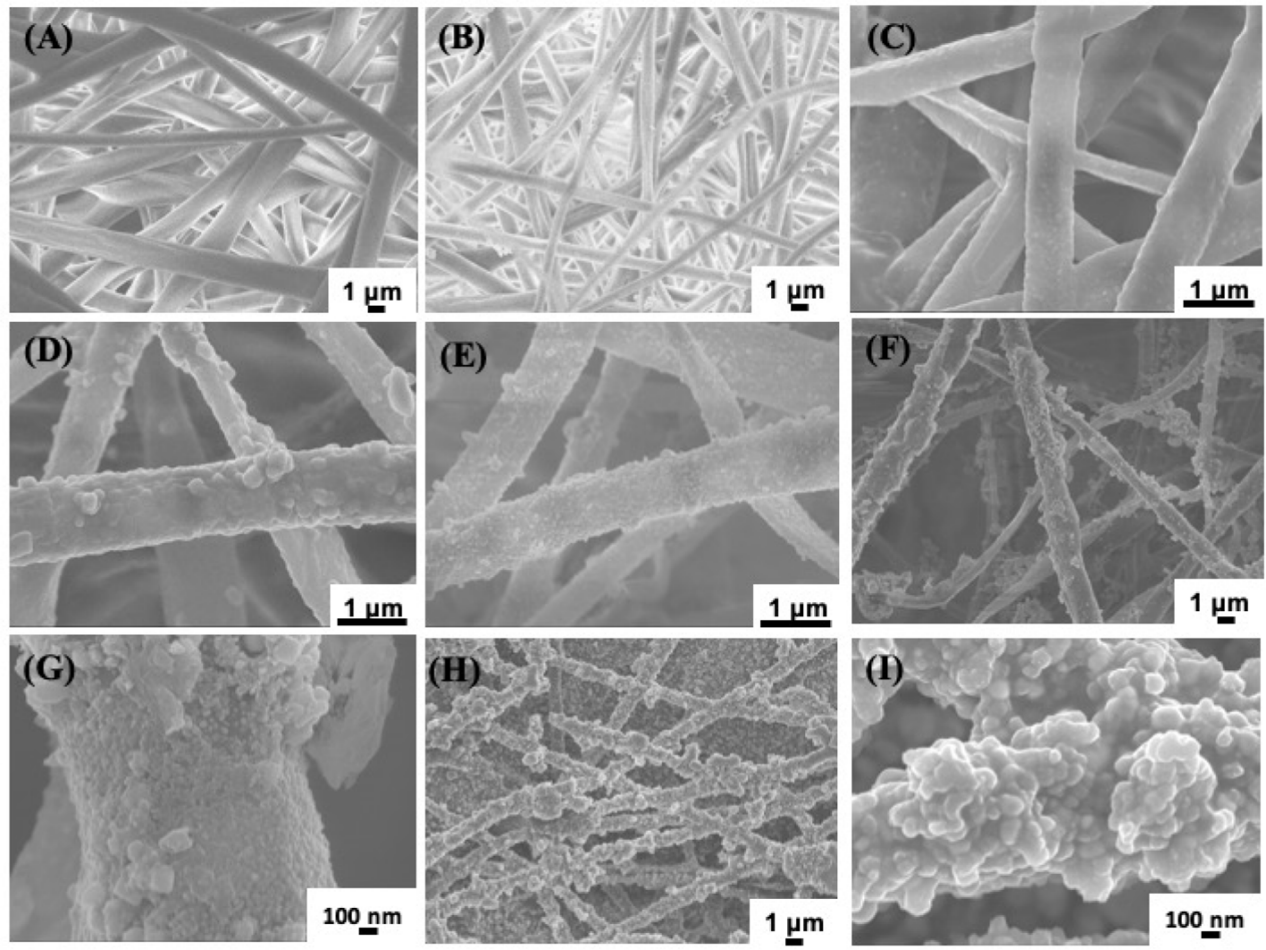
SEM images of different samples. Poly(L-lactide) (PLLA) **(A)**, PLLA@PDA **(B)**, PLLA@Ag1 **(C)**, PLLA@Ag3 **(D)**, PLLA@Ag6 **(E)**, PLLA@Ag9 **(F**, **G)**, and PLLA@Ag 24 **(H**, **I)**.

Electrospinning has been a versatile technique to prepare nonwoven membranes used for bone tissue engineering or bone regenerative (Ding et al., 2019; Feng et al., 2019). In this work, we firstly prepared PLLA membrane *via* electrospinning method, and then mussel inspired method was used to prepare silver coated PLLA membrane. To observe the change of electrospun fibers, SEM were used to observe the morphology of different samples. The PLLA membranes show porous structure, and the fibers surface are smooth (**Figure 1A**). When the PLLA membranes were immersed in basic dopamine solution, dopamine will self-polymerize and form polydopamine (PDA) on the surface of PLLA fibers. As shown in **Figure 1B**, the morphology of PLLA@PDA membrane are almost the same with that of PLLA (**Figure 1A**). The PDA layers have strong interaction with metal ions and can also reduce silver ion (Ag^+^) to AgNPs (Wu et al., 2015; Wang et al., 2017). When PLLA@PDA was immersed in AgNO_3_ solution, the Ag^+^ will bind to PDA and be reduced to AgNPs, with the increase of reaction time, the AgNPs particle size or amount will increase. As shown in **Figure 1C**, only a few particles were anchored on the surface of PLLA fibers, when the reaction time was 3 h, much more AgNPs appeared on the surface of fibers. If the reaction time was 6 hours, it can be seen that, the surface of PLLA fibers were nearly coated with AgNPs. After 24 h, the AgNPs will aggregate heavily on the surface of PLLA fibers (**Figures 1H, I**).

**Figure 2:**
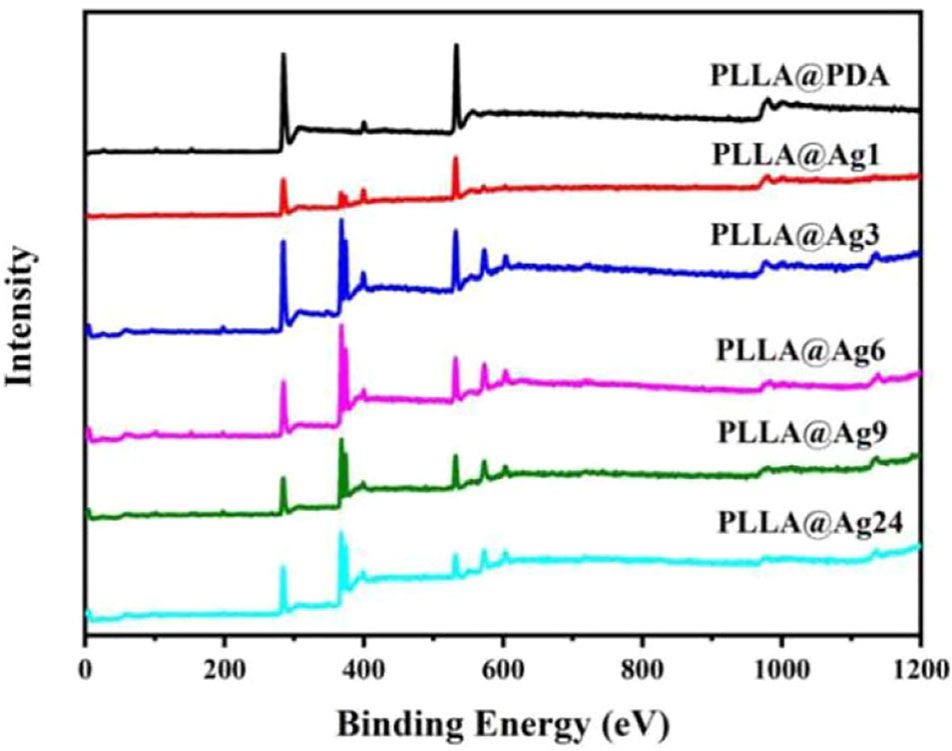
X-ray photoelectron spectrometer (XPS) curves of poly(L-lactide) (PLLA)@PDA, PLLA@Ag1, PLLA@Ag3, PLLA@Ag6, PLLA@Ag9, PLLA@24.

To further analyze the change of surface composition of PLLA membrane, XPS measurements were carried out. The PLLA surface consist of C and O elements. When PDA were coated on the surface of PLLA fibers, a new element, N will appear, as shown in **Figure 2**, in the spectrum of PLLA@PDA, a weak peak centered at 399.0 eV was found, which was the characteristic signal of N element, strongly confirming the formation of PDA coating on PLLA fibers. When AgNPs were formed on the surface of PLLA@PDA, XPS will show signals of AgNPs, as shown in **Figure 2**, all the PLLA@Ag samples showed two individual peaks at 368.7 eV and 374.7 eV with a spin-orbit separation of 6 eV, which proves the existence of the AgNPs. According to the quantitative results of XPS, the Ag Content (At. %) of PLLA@Ag1, PLLA@Ag3, PLLA@Ag6, PLLA@Ag9, and PLLA@Ag24 membranes were 1.6%, 4.9%, 7.9%, 8.2%, and 15.4%, respectively, implying that the AgNPs’ content can be easily tuned by tuning the reaction time.

**Figure 3:**
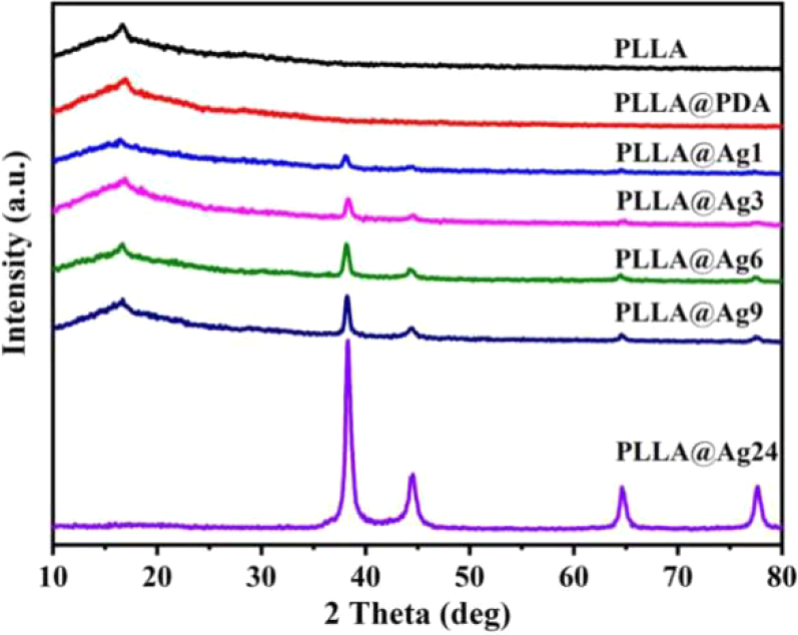
X-ray diffractometer (XRD) patterns of poly(L-lactide) (PLLA)@PDA, PLLA@Ag1, PLLA@Ag3, PLLA@Ag6, PLLA@Ag9, PLLA@24.

The XRD patterns of various samples are shown in **Figure 3**. As for neat PLLA electrospun nanofibers membrane, a weak peak appeared at 16.6°, corresponding to (200)/(100) planes, however, PLLA is a semi-crystalline polymer, most of the fibers are in amorphous state (**Figure 3**). The XRD pattern of PLLA@PDA nanofibers has no difference with that of PLLA, suggesting that dopamine self-polymerization does not affect the structure of PLLA nanofibers. When PLLA@PDA was immersed into the solution of AgNO_3_, Ag^+^ will be quickly chelated, then the Ag^+^ will be reduced to Ag^0^ and formed AgNPs on the surface of PLLA fibers. As shown in the XRD patterns (**Figure 3**), new sharp peaks appear at 38.26°, 44.37°, 64.65°, and 77.61° in the patterns of all PLLA@Ag samples, and these peaks are assigned to the characteristic crystalline planes of (111), (200), (220), and (311) of the *fcc* structured Ag, correspondent with the data of JCPDS No. 04-0783 (2θ = 38.096°, 44.257°, 64.406°, and 77.452°). With the increase of reaction time (from 1hour to 24 h), the intensity of peaks increased, demonstrating that the amount of AgNPs is time-dependent. Furthermore, these results can clearly verify that it is an effective method to prepare silver coated PLLA membranes.

### Antibacterial Activity

**Figure 4:**
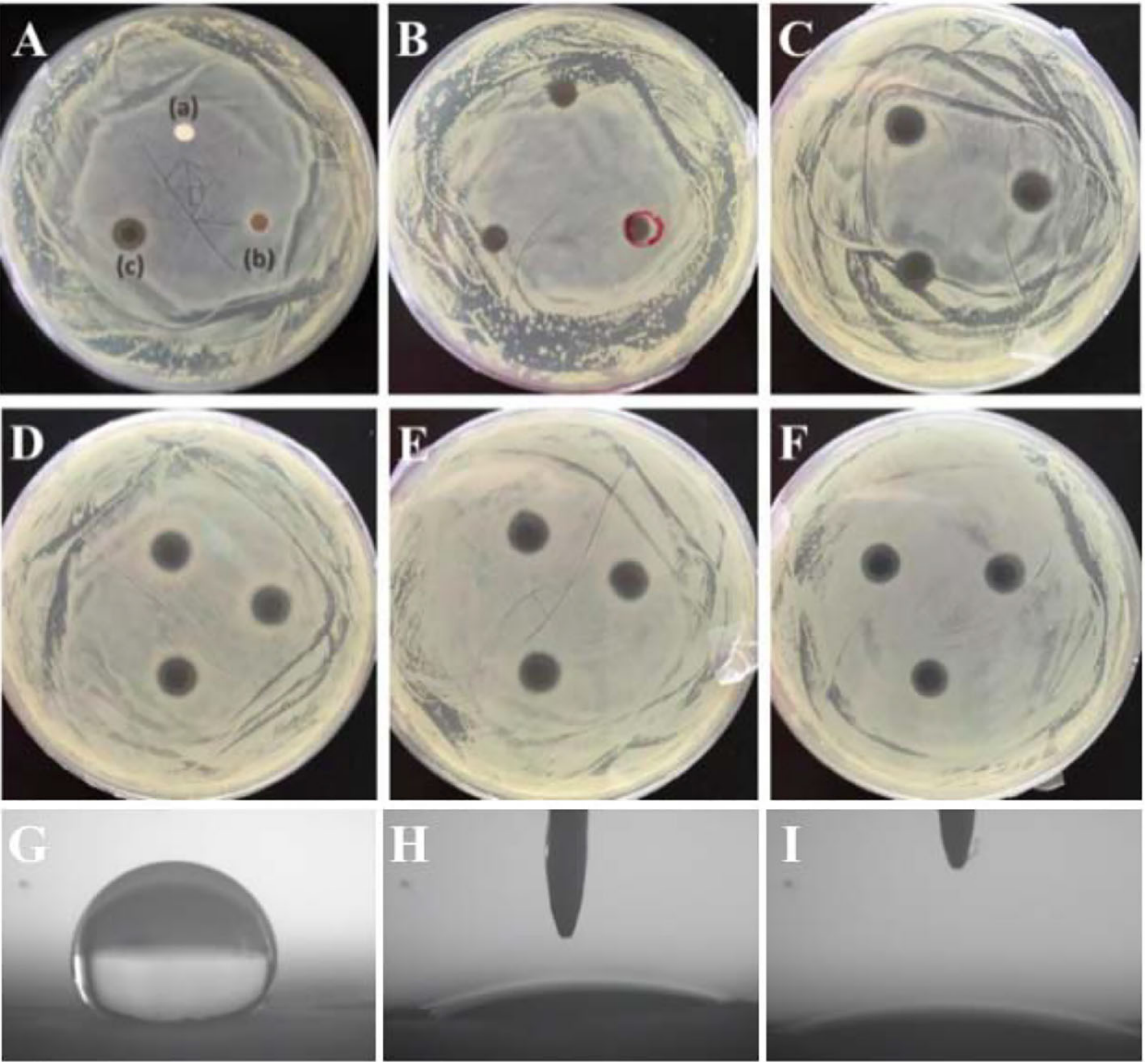
**(A–F)** showed the antibacterial activity of electrospun membranes against *S. aureus*. poly(L-lactide) (PLLA) (A-a), PLLA@PDA (A-b), PLLA@Ag24 (A-c). **(B–F)** are the corresponding results of PLLA@Ag1 **(B)**, PLLA@Ag3 **(C)**, PLLA@Ag6 **(D)**, PLLA@Ag9 **(E)**, and PLLA@Ag24 **(F)**, respectively. **(G**–**I)** are the contact angle images of PLLA, PLLA@PDA and PLLA@Ag 24, respectively.

Antibacterial property is very critical for GBR membranes. Concerning the perspective of PLLA membrane used as GBR membrane, it is an valuable thing to endow PLLA membrane with antibacterial property. Due to the excellent antibacterial property of AgNPs, the prepared PLLA@Ag must have antibacterial property. Up to now, many researches have proved that AgNPs or AgNPs coated materials showed excellent antibacterials to both the Gram-positive *S. aureus* and Gram-negative *E. coli* (Wu et al., 2015; Wang et al., 2017). On considering that *S. aureus* is an important bacterial related with stomatologic problem, such as maxillofacial infections and peri-implantitis (Persson and Renvert, 2014), and thus in this work, *S.aureus* was selected as the bacterial representative to assess the antibacterial activity *via* observation the growth of *S. aureus* on the agar plate. As shown in **Figure 4A**, Bacterial inhibition zones are clearly observed around PLLA@Ag membrane (PLLA@Ag 24 was used in **Figure 4A**), whereas, PLLA and PLLA@PDA did not show any inhibition zone (**Figure 4A** a, b). **Figures 4B–F** showed the bacterial inhibition effect of PLLA@Ag1, PLLA@Ag3, PLLA@Ag6, PLLA@Ag9, and PLLA@Ag24, respectively, and the results clearly showed that the prepared PLLA@Ag have effective antibacterial effect. PLLA@Ag1 membrane presented only 7.19 ± 0.18 mm diameter and kept less than 7 days (**Table 1**). By contrast, all the other PLLA@Ag membranes showed much better antibacterial activity. It is worth mentioning that the inhibition zones are still obvious even after 14 days except the results for PLLA@Ag1. However, from the results of PLLA@Ag3, PLLA@Ag6, PLLA@Ag9, and PLLA@Ag24, the increase of AgNPs amount did not result in higher inhibition zones (**Figures 4C–F**), while this was consistent with bactericidal efficiency results calculated from the modified colonies counting tests (**Table 1**). All the bactericidal efficiency of PLLA@Ag membranes were more than 95% against the targeted bacteria, except PLLA@Ag1 membranes (only 32%). Hence, it can be concluded that the incorporation of AgNPs endowed PLLA membrane antibacterial property.

**Table 1:** The average diameter of inhibition zone and bactericidal efficiency of different samples.

Sample	Inhibition zone diameter (mm)				Bactericidal efficiency (%)
	1 day	3 days	7 days	14 days	
PLLA@Ag1	7.19 ± 0.18	7.13 ± 0.11^*^	0	0	32
PLLA@Ag3	8.84 ± 0.34	8.45 ± 0.27	8.42 ± 0.19	8.59 ± 0.37	97.9
PLLA@Ag6	9.07 ± 0.21	8.93 ± 0.21	8.97 ± 0.04	8.93 ± 0.13	98.3
PLLA@Ag9	8.92 ± 0.22	8.72 ± 0.06	8.54 ± 0.12	8.96 ± 0.17	96.6
PLLA@Ag24	9.50 ± 0.09	8.97 ± 0.07	9.02 ± 0.06	8.61 ± 0.16	97.0

Although the mechanism for AgNPs antimicrobial activity is still controversial, it is no doubt that the presence of AgNPs could act as Ag^+^ reservoir, and provide continuously a high enough concentration of silver species in their surroundings to maintain its antibacterial activity for several days (Le Ouay and Stellacci, 2015). Herein, because of the strong interactions between AgNPs and PDA coating, the AgNPs would not easily detached from the PLLA matrix, and thus the prepared PLLA@Ag can have a long-time antibacterial activity.

Up to now, commercial polylactide membrane (including PLLA and PDLLA) and their blend with other polymers or functional agents have been used as GBR membrane in clinic (Wang et al., 2016), however, the bacterial infections have been a challenge for commercial GBR membrane. And thus, the prepared PLLA@Ag membrane showed some advantages over the pure PLLA membrane. Furthermore, the surface of materials may have great affection on their properties, many methods have been designed to modify polymer membranes used as GBR membrane, such as antibiotic coating, polymer coatings (Florjanski et al., 2019). In this work, we prepared AgNPs coated PLLA membrane *via* a mild mussel inspired method, the most important results maybe endowing the antibacterial properties to PLLA. Besides, the surface wettability were also improved, as shown in **Figure 4**, the surface of PLLA membrane was hydrophobic (**Figure 4G**), while PLLA@PDA (**Figure 4H**) and PLLA@Ag24 (**Figure 4I**) are hydrophilic. In addition, the PDA may also promote the biomineralization of hydroxyapatite. And thus, compared with the PLLA membrane, the PLLA@Ag membrane have many advantages. However, the potential *in vivo* toxicity of PLLA@Ag should be further investigated.

### Cytotoxicity Test

**Figure 5:**
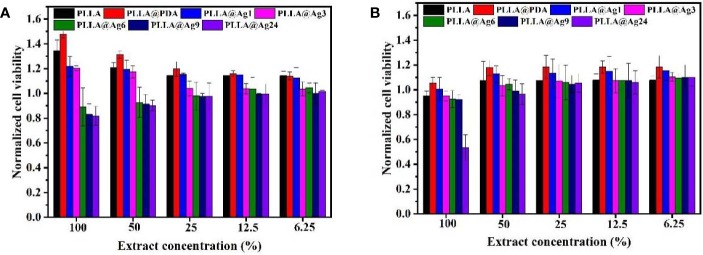
Cell viability of different samples against MC3T3 Cells **(A)** and L929 Cells **(B)**.

PLLA is a biocompatible polymer, while PDA is also biocompatible, and thus the PDA coated PLLA fiber membrane also showed low toxicity. As shown in **Figures 5A, B**, the cell viability of both MC3T3 and L929 cell line treated with different amount of PLLA@PDA are much better, with a cell viability more than 100%. Generally, AgNPs showed low toxicity and have been widely used as antibacterial agent. When AgNPs were immobilized on the surface of PLLA@PDA fibers, it can combine the biocompatibility of PLLA@PDA and antibacterial property of AgNPs, and thus PLLA@Ag can work as antibacterial GBR membrane. Besides, the biocompatibility of PLLA@Ag is an important factor that determines the application of PLLA@Ag. As shown in **Figure 5**, the cell viability of MC3T3 on PLLA@Ag1 and PLLA@Ag3 are about 120%, respectively, however, with the increase of AgNPs amount, the samples will show toxicity. For example, the MC3T3 cell viability of PLLA@Ag6, PLLA@Ag9, and PLLA@Ag24 membranes were lower than that of PLLA@Ag1 and PLLA@Ag2 (**Figure 5A**). To further test the biocompatibility of PLLA@Ag membrane, L929 cells were used, it is interesting to find that almost all the nanofiber membranes presented good cytocompatibility on L929 (**Figure 5B**). However, when the sample concentration was too high, for example, when 100% extract were used, the cell viability of PLLA@Ag24 was only 53.32% (**Figure 5B**), much lower than that of other samples. From results in **Figure 5**, it can be concluded that the biocompatibility of PLLA@AgNPs membrane are very good, the samples prepared in this work may have potential application as GBR membrane.

## Conclusions

In this work, AgNPs was immobilized on the surface of PLLA nanofibers *via* the simple mussel inspired method to obtain antibacterial PLLA nanocomposites (PLLA@Ag). The *in vitro* test showed that the PLLA@Ag have excellent antibacterial property, more interestingly, the PLLA@Ag can have long term antibacterial effect, even after 2 weeks, the prepared sample can still prevent the growth of bacterial. The cytotoxcity test showed that the PLLA@Ag sample showed little toxicity, when MC3T3 cell was used, all the samples showed a little toxicity when compared with PLLA. However, when L929 cells were used, the samples showed comparable biocompatibility with PLLA.

The method used in this work was simple and versatile, and may be used to immobilize AgNPs on other materials, besides, due to the various *in vivo* application of PLLA, such as GBR membrane, the PLLA@Ag may also have broad applications as implants that can prevent infections.

## Data Availability Statement

The datasets generated for this study are available on request to the corresponding authors.

## Author Contributions

JWe and LL proposed this project. JWa prepared all the materials. LZ carried out the antibacterial test and cytotoxicity test. XZ and RW help to analyze the results. JWa and JWe wrote the manuscript. JWa and LZ have the same contribution to this work.

## Funding

This research was funded by the National Natural Science Foundation of China (Nos. 51663017 and 81660444), Jiangxi Key Research and Development Program (NO.20181BBG78014), Natural Science Foundation of Jiangxi Province (NO.20171BAB205050).

## Conflict of Interest

The authors declare that the research was conducted in the absence of any commercial or financial relationships that could be construed as a potential conflict of interest.
